# The WHO-ITU MyopiaEd Programme: A Digital Message Programme Targeting Education on Myopia and Its Prevention

**DOI:** 10.3389/fpubh.2022.881889

**Published:** 2022-05-26

**Authors:** Stuart Keel, Pirindha Govender-Poonsamy, Alarcos Cieza, Hannah Faal, Ian Flitcroft, Kate Gifford, Mingguang He, Rajiv Khandekar, Kovin Naidoo, Matt Oerding, Kyoko Ohno-Matsui, Silvio Mariotti, Christine Wildsoet, James S. Wolffsohn, Tien Y. Wong, Sangchul Yoon, Andreas Mueller, Rosie Dobson

**Affiliations:** ^1^Department of Noncommunicable Diseases, World Health Organization, Geneva, Switzerland; ^2^African Vision Research Institute, University of KwaZulu Natal, Durban, South Africa; ^3^Centre for Eye Research, Dublin, Ireland; ^4^Myopia Profile Pty, Ltd, Brisbane, QLD, Australia; ^5^Department of Optometry and Vision Sciences, University of Melbourne, Melbourne, VIC, Australia; ^6^King Khaled Eye Specialist Hospital, Riyadh, Saudi Arabia; ^7^EssilorLuxottica, Paris, France; ^8^Global Myopia Awareness Coalition, Boulder, CO, United States; ^9^Department of Ophthalmology and Visual Science, Tokyo Medical and Dental University, Tokyo, Japan; ^10^School of Optometry, University of California, Berkeley, Berkeley, CA, United States; ^11^Optometry and Vision Science, Aston University, Birmingham, United Kingdom; ^12^Singapore National Eye Centre, Duke-NUS Medical School, National University of Singapore, Singapore, Singapore; ^13^Dept of Medical Humanities and Social Sciences, Yonsei University, Seoul, South Korea; ^14^National Institute for Health Innovation, University of Auckland, Auckland, New Zealand

**Keywords:** myopia, mHealth, public health, digital health, behavior change

## Abstract

The objective of this paper is to provide an overview of the World Health Organization - International Telecommunication Union MyopiaEd programme - a digital message programme targeting education on myopia and its prevention. The development of the MyopiaEd programme included 4 key steps: (1) Conceptualization and consultation with experts in the field of myopia, mHealth and health behavior change; (2) Creation of SMS message libraries and programme algorithm; (3) Review of the message libraries to ensure relevance to the target audience; and (4) Pre-testing amongst end-user groups to ensure that the design of the programme and the message content were understandable. After reviewing the available evidence and considering input of the experts, the aims, end users and key themes of the programme were finalized. Separate SMS-adapted message libraries were developed, reviewed and pre-tested for four target end-user groups; (1) general population involved in the care of children (2) parents or caregivers of children with myopia; (3) adolescents with myopia; and (4) adults with myopia. The message libraries are part of a comprehensive toolkit, developed through a consultative process with experts in digital health, to support implementation within countries. The development of the MyopiaEd programme aims to provide a basis for Member States and other stakeholders to develop, implement and monitor large-scale mHealth programmes. It is aimed at raising awareness of good eye care behaviors and addressing common reasons for non-compliance to spectacle wear. The next steps will involve adapting and evaluating the MyopiaEd programme in selected settings.

## Introduction

Uncorrected myopia is a leading cause of vision impairment and poses a considerable financial burden on countries, with an estimated annual global productivity loss of US$ 244 billion (1). To further confound this problem, the prevalence of myopia is projected to increase substantially in the coming decade, with 3.36 billion people estimated to be impacted by 2030. (2) During the same period, the number of people with high myopia, an emerging cause of irreversible blindness, is projected to impact over 500 million. Although refractive correction provides an effective means of correcting myopia, compliance with spectacle-wear among children and adolescents is often suboptimal, commonly attributable to misconceptions and stigma (3). In addition, awareness of the risk factors and symptoms of myopia are low and may prevent or delay children from receiving a formal eye examination.

Growing evidence among child populations strongly implicates lifestyle risk factors, including intensive near vision activity (as a risk factor) and longer time spent outdoors (as a protective factor), in the onset and progression of myopia during childhood (4). Interventions targeting these lifestyle factors offer the possibility of reducing the risk of developing high myopia and its related potentially blinding complications later in life. To this end, large-scale programmes and policies have been established within countries with a high prevalence of myopia, aimed at myopia prevention through increased time spent outdoors among children (5, 6).

In October 2019, WHO launched the World Report on Vision which highlights the importance of preventive strategies for eye conditions (2). A key recommendation of the report was to raise general awareness and engage and empower people and communities (2). In line with this recommendation, WHO recognizes the vital role education campaigns play in the management of myopia and its associated complications, while also improving education of good eye care behaviors (e.g., the importance of regular eye examinations) and addressing common reasons for non-compliance to spectacle wear. However, research shows that in many countries, awareness of myopia is low (7, 8). Cognisance is also given to the possible deleterious effect of COVID-19 lockdown measures on myopia amongst children, with research indicating an increase in myopia incidence and progression attributed to less time spent outdoors due to home confinement and a substantial increase in near work activities such as online learning (9–11).

To facilitate countries in enhancing their domestic services for myopia education and prevention, WHO and ITU have developed the “Be He@lthy, Be Mobile” (BHBM) programme for myopia. The BHBM programme uses mobile technology for health (mHealth) to address a range of non-communicable diseases and health issues such as diabetes, dementia, aging, and tobacco consumption. In this case, building on already acquired experience in mHealth, and in collaboration with an international group of experts in the field of myopia and behavioral science, an mHealth programme for myopia – MyopiaEd – has been developed. The objective of this paper is to provide an overview of the MyopiaEd programme, including the development process, and outline the next steps.

## Methods

### Development and Design of the MyopiaEd Programme

**Figure 1:**
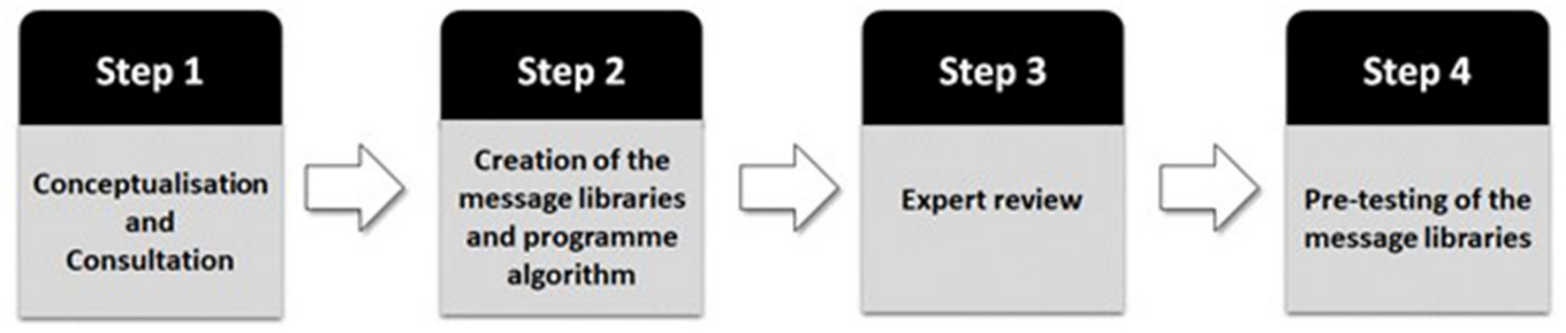
Stages of development of the MyopiaEd programme.

The development of the MyopiaEd programme was aligned with published development frameworks (12, 13) with a focus on implementation, use of behavioral change theory, and involvement of the target population. The development process followed a stepwise process with the involvement of different stakeholders (Figure 1).

#### Step 1. Conceptualization and Consultation

The WHO was responsible for the overall coordination of the project as well as technical and developmental work.

An Informal Expert Group (IEG), comprising experts in myopia and health behavior change, purposively recruited from all six WHO regions, was established at the project outset to provide technical input throughout the development of the MyopiaEd Programme. Initial technical consultations were held (22–23 October 2020) with WHO offices, including those from the Vision and Eye Care Programme and Digital Health Department, and the IEG with the principal objective being to agree on the scope of the MyopiaEd programme, including the context, purpose, end users and key topic themes to be covered.

In preparation for the IEG consultations, WHO offices identified existing evidence (e.g., systematic reviews, randomized control trials, white papers) (14–23) on a range of topics (e.g., spectacle compliance, time spent outdoors and near work-related parameters), as the basis for identifying key topic themes to be covered in the MyopiaEd programme. This evidence and proposed themes were summarized during the consultation, where IEG members provided technical input to WHO on the nature of the related message content within each theme, with consensus being the endpoint in all cases (14–23).

#### Step 2. Creation of the Message Libraries and Programme Algorithm

Following the initial consultation period with the IEG to finalize the key themes and end-users, an expert in health behavior change (RD), who previously led the development of message libraries for other Be He@lthy Be Mobile programmes, (24, 25) drafted separate MyopiaEd message libraries for each end user group (26). Message content was written with a global perspective and with the understanding that the messages may need to be adapted for use by specific countries. Behavior change techniques were used to underpin each of the messages. Each message was categorized into one of 4 domains: Motivation, Support, Information, or Reminders. Messages were designed to be clear and direct, offering practical and relevant advice, in simple language. Messages were designed to be positively (gain/benefit) framed, with a focus and emphasis on the benefits of action.

Suggested algorithms for the programme were also developed to guide the delivery of the messages. Based on the experience of WHO mHealth programmes, expert review and end user feedback, suggestions regarding the format, the timing of the programme, and frequency of messages to be delivered were made.

The expert in health behavior change had access to the following reviews and information to assist in informing the initial draft of the MyopiaEd message libraries and algorithms:

(i) A scoping review commissioned by WHO to collate and synthesize the evidence on the use of mHealth interventions in eye care, where they provide information to raise awareness about services, provide condition-specific information or encourage individuals to adhere to a treatment or to attend an appointment.
(ii) A review of existing social media campaigns on myopia and collation of existing messages used in myopia-related awareness or health promotion campaigns. The search was conducted on News, blogs, websites (governmental or non-governmental organizations) and publications, together with a focused social media search using the Sprinklr^®^ platform which listens to mentions across more than 20 social channels.
(iii) The results of unpublished end user qualitative and quantitative research conducted by the Global Myopia Awareness Coalition (GMAC). This research explored various strategies and campaigns on myopia education for key stakeholders including parents, children and healthcare providers in the United States (27).

#### Step 3. Expert Review

The initial message libraries and associated algorithm underwent two rounds of expert review. This included (i) review by experts in the field of myopia to ensure that the messages were clinically correct and evidence-based; and (ii) review by experts in behavior change from the WHO Behavioral Insights team (Geneva) and WHO Regional Office digital health advisors to ensure that the messages were relevant to the target audience. Following each stage of expert review, the message libraries were progressively updated to incorporate experts' feedback.

#### Step 4. Pre-testing of the Message Libraries

The purpose of pre-testing the message libraries was to ensure that both the design of the programme and the draft message content were understandable and acceptable to an English-speaking target audience.

Convenience targeted sampling was used to recruit participants for the pre-testing to ensure representation from all potential types of end users. During the pre-testing, the proposed programme was described, and a range of messages was shown. Participants were asked to provide feedback on aspects of the proposed programme (e.g., programme duration, target users, frequency of messaging), as well as on the individual messages (e.g., clarity, tone, content). Thirteen sessions of pre-testing were carried out by a trained interviewer in-person or over videoconferencing, according to the participant's preference. Feedback from participants were summarized by the interviewer and common themes were identified using a simple, general inductive thematic approach. Based on the feedback received during pre-testing, additional changes were made to the message libraries.

## Results

### Key Outcomes of the Informal Expert Group Consultation

The key outcomes and discussion points from the IEG consultation (October 2020) for each of the proposed themes of the MyopiaEd programme are summarized in Table 1.

**Table 1:** A summary of the key outcomes of the WHO consultation on the proposed themes of the MyopiaEd Programme.

**Theme of messages**	**Key outcomes and discussion points**
General myopia education and misconceptions	•It was agreed that the messages on general myopia education would cover the key areas of (i) what is myopia?; (ii) prevalence; (iii) causes; and (iv) warning signs and potential long term consequences of myopia. •It was acknowledged that some myopia misconceptions are very culturally specific. Given the MyopiaEd toolkit is aimed at a global audience, these misconceptions should be incorporated into the message libraries during the adoption phase that will happen at an individual country level. •It was agreed that the messages on global myopia misconceptions would focus on the key areas of: (i) wearing spectacles makes your child's myopia worse; (ii) there is nothing you can do to prevent myopia or vision loss from myopia; (iii) myopia only affects children; and (iv) myopia is just a spectacle/vision issue and not an eye health issue
Regular comprehensive eye exams	•It was agreed that messages promoting eye examinations amongst all population end-users are important. •While it was not considered feasible to make a recommendation on age and frequency of examinations at a global level, it was recommended that the toolkit should aim to be specific and instructional, to provide the end-user with actionable items for change. Therefore, the message libraries should promote inclusion of age and frequency when message content is adapted at a country level to be aligned with other eye health programme guidance within the specific country.
Time spent outdoors	•There was general agreement that the evidence is sufficient to promote time spent outdoors as a key theme in the MyopiaEd programme. •It was noted that the evidence is stronger for primary prevention (i.e., reducing the incidence) than secondary prevention (i.e., slowing progression to reduce the risk of high myopia). Therefore, messages promoting increased time spent outdoors are used most frequently in the MyopiaEd message library targeting the general population involved in the care of children without myopia. However, given the safety of the intervention, and the potential broader benefits for physical and mental health, it was agreed that messages promoting time spent outdoors should also be included in the message libraries targeting young people with myopia, albeit in a reduced frequency. •While it was acknowledged that further research is required in order to be able to provide precise recommendations on the amount of time per day, the IEG felt strongly that an amount of time per day should be specified in order to provide the end-user with actionable items for change. To this end, it was suggested that the evidence is sufficient to at the least include recommendations for a minimum daily time spent in outdoor leisure activities and, based on the evidence, 90 min was proposed (28–30). It was acknowledged that the message content should be adapted as further evidence becomes available. •Other key considerations for messages promoting increased time outdoors included: (1) the need to consider sun-protection in some latitudes; and (2) cultural commitment to educational success and weather as potential barriers. To this end, the messages aim to avoid people misinterpreting them as “anti-education”; rather the focus should be on encouraging more time outdoors during leisure time. Messages also aim to avoid exposing children to weather-related health risks.
Education, near work-related parameters, screen time	•There was general agreement that the evidence is sufficient to include time spent on near-work related activities as a theme in the MyopiaEd Programme. This was based on the findings of recent systematic reviews of cross-sectional studies that have concluded that more time spent on near work activities was associated with higher odds of having myopia. However, the paucity of evidence from RCTs on this topic, as well as the difficulty to conduct such research, was acknowledged. •It was agreed that the evidence on the relationship between personal digital devices use and myopia onset or progression is mixed and not yet comprehensive. However, many members of the IEG felt strongly that to *not* include messages on this theme would be a missed opportunity, particularly given that evidence strongly implicates device use and (i) other eye-related conditions, e.g., dry eye-related complications; and (ii) other health issues, e.g., mental health. Therefore, it was suggested that digital devices could be included within the message content as an example of a near-work activity. •As with outdoor activity, it was suggested that negative messaging on reading and education should be avoided. Rather the focus should be on encouraging changes in behavior during leisure time.

### Scope of the MyopiaEd Programme

After reviewing the available evidence and considering the feedback of IEG members, and individuals from related WHO departments, the aims, end users, key themes and algorithm (i.e., format, frequency and duration) of the MyopiaEd programme were finalized (Table 2).

**Table 2:** Overview of key aspects of the MyopiaEd programme (31).

**Aim**	**1. To support behavior change that contributes to delaying the onset, and slowing the progression, of myopia 2. To improve awareness and health literacy of the importance of regular eye examinations and spectacle compliance among children and adults with myopia**
**End users**	**1. General population involved in the care of children, including general health workers and educators 2. Parents or caregivers of children with myopia 3. Adolescents with myopia 4. Adults with myopia**
Themes of the messages^*^	**1. General education on myopia: the causes, warning signs and misconceptions 2. Lifestyle behavior changes, including time spent outdoors and near-work related parameters, that can reduce the risk of high myopia and its complications 3. Importance of regular comprehensive eye examinations 4. Importance of compliance with refractive correction**
**Message format**	**Messages have been designed for one-way SMS (text message) delivery, but are appropriate for delivery via other modalities, including app messaging and social media**.
**Message frequency**	**The programme starts at a higher frequency and decreases over time (32). Repetition of key messages is important to ensure that they are understood, behavior change is supported and then maintained. Where repetition of key messages occurs, these are spaced to reduce the likelihood of user boredom**.
**Duration of the programme**	**The suggested algorithm for the message library aimed at the general population is 12 months in duration. For the remaining message libraries developed for end-user groups who already have myopia, the suggested algorithms are approximately 6 months in duration. This duration was chosen based on evidence showing that complex change in health behavior takes 6 months to be habitually incorporated into a person's lifestyle (33, 34)**.

* *While messages on general myopia education (such as prevalence, and potential long-term consequences) apply to all population target end users, the themes of other messages vary according to the profile of the individual*.

### Message Libraries and Toolkit to Support Implementation

Key changes made to the message libraries following this peer review process, coupled with an overview of the feedback received during pre-testing of the message libraries, have been summarized in Supplementary File 1.

The resultant message libraries for the four target end-user groups, and an accompanying BHBM toolkit containing operational guidance and resources to support the implementation, can be found at the WHO webpage (26).

## Discussion

The WHO-ITU MyopiaEd programme provides a basis to support countries and other stakeholders to develop, implement and monitor large scale mHealth programmes aimed at (i) improving awareness of the importance of regular eye examinations and spectacle compliance, and (ii) supporting behavior change that may delay the age of onset, and slow the progression of myopia.

Traditionally, interventions aimed at health promotion and prevention in the field of eye care have received less attention and investment than those for treatment. However, the growing evidence implicating lifestyle risk factors in the onset and progression of myopia, coupled with the known impact of uncorrected myopia on academic performance and the need to address common reasons for non-compliance with spectacle wear, provide a strong rationale for educational campaigns targeting both those at risk of developing myopia and those who already have myopia. A text message-based programme, such as that described in this paper, offers a solution to reaching large audiences at low cost. While not extensive, literature on the use of mHealth messaging in eye care shows promising results for improving adherence to treatment of chronic eye conditions, (35, 36) increasing rates of attendance at eye care facilities (37–39) and, more recently, behavior modification for the prevention of myopia (40).

Similar to the other WHO BHBM programmes (41) the MyopiaEd programme is intended for implementation by government officials, academics, and other in-country partners (e.g., non-government organizations) who are involved in mHealth, or other health promotion, programmes. As mentioned, the message libraries (26) are accompanied by a comprehensive BHBM toolkit (31) to support the implementation of the programme within individual countries. Specifically included are introductions and considerations specific to the development of a workplan for an MyopiaEd programme, the role of different stakeholders, guidance for adapting the messages to the local context, selection and implementation of the best technology to deliver the programme, strategies for promotion and retention, and guidance and resources to support monitoring and evaluation (31). Of note, effective promotion will be essential to recruit users to the MyopiaEd programme and enabling them to subscribe in a convenient manner. To this end, it is recommended that multiple engagement channels are used (e.g., social media, SMS, community and civil society meetings, and various other gatherings). On enrolment to the programme, a pre-screening questionnaire will be used to select the most relevant message library for each user based on their characteristics.

A number of key actions are required prior to large scale implementation of the MyopiaEd programme (31, 41, 42). Firstly, the current MyopiaEd message libraries (26) have been written from a global perspective, and, although pre-tested in a high-income English-speaking target audience, it is acknowledged that many of the social environments in which these messages may be deployed will have difference characteristics. Therefore, prior to implementation, the message libraries will need to be translated, adapted, and/or additional content developed, based on the social or cultural context in each country or setting. For example, references to contact lenses and other treatment options could be added where available and accessible (the current message library refers to spectacles as the main form of correction), references to sun protection should be added where applicable in messages encouraging time spent outdoors, and the specific details of the recommended age of first eye examination, and frequency of eye examinations based on country-level guidelines should be added (taking into account national health service provision and current screening programmes). Adapted content will enable users to relate to and implement the strategies for behavioral change and may lead to higher retention of users. Local experts and target users should guide the adaptation process, with any new information being strictly evidence-based. Secondly, the next stage of the project will involve evaluating the MyopiaEd programme in selected settings to determine the impact of the programme, facilitate course correction, and make the case for expansion. Evaluation will focus on key outcome indicators, including changes in knowledge or behaviors that have occurred as a result of the programme. Lastly, acknowledging that evidence in the field of myopia is subject to change, future work will involve periodically reviewing, updating and refining the MyopiaEd message libraries.

The short-term objectives of the MyopiaEd programme are to (i) improve population awareness and health literacy on myopia; (ii) contribute to eliciting modifications in behavior of the population (i.e., care seeking, reduced time spent on near work activities during leisure time and increased time spent outdoors in children); and (iii) address misconceptions and stigma to positively impact on the willingness to wear and/or time spent wearing spectacles among children and adolescents. It is important to emphasize that the MyopiaEd is not intended to be conducted in isolation, but rather it should be complementary to existing and emerging screening and clinical interventions, policies and awareness related to general health (e.g., obesity control through physical activity), myopia and eye health in countries. If successful in the long-term, these interventions along the continuum of care have potential to reduce the incidence of (i) childhood myopia, (ii) high myopia and (iii) irreversible vision impairment due to myopia. As key underpinnings of an effective programme, robust monitoring and evaluation strategies are planned to assess the programme activities, outputs, and outcomes and thereby its overall performance (43).

In conclusion, it is the intention that the MyopiaEd programme will provide a basis to strengthen countries' efforts to develop sustainable, cost-effective, and acceptable activities to support education on myopia and its prevention. Of importance, it is recommended that the programme be implemented as part of an existing national or regional digital health or mHealth programme (where available) to ensure optimization of available resources. The next phase of this project will focus on country adaptation for implementation, and evaluation in selected settings.

## Data Availability Statement

The datasets presented in this study can be found in online repositories. The names of the repository/repositories and accession number(s) can be found in the article/Supplementary Material.

## Ethics Statement

Ethical review and approval was not required for the study on human participants in accordance with the local legislation and institutional requirements. Written informed consent from the participants' legal guardian/next of kin was not required to participate in this study in accordance with the national legislation and the institutional requirements.

## Author Contributions

SK, AC, and AM conceived the project. RD conducted the user-testing. SK prepared the first and subsequent drafts of this manuscript, following co-author review. All authors reviewed and approved the submission of the final manuscript.

Members of the WHO BEHAVIORAL INSIGHTS team Elena Altieri and Sarah Elaraby.

## Author Disclaimer

The views expressed in this paper are those of the authors and do not necessarily reflect the views of WHO.

## Conflict of Interest

KG was employed by Myopia Profile, Pty, Ltd. KN was employed by EssilorLuxottica. The remaining authors declare that the research was conducted in the absence of any commercial or financial relationships that could be construed as a potential conflict of interest.

## Publisher's Note

All claims expressed in this article are solely those of the authors and do not necessarily represent those of their affiliated organizations, or those of the publisher, the editors and the reviewers. Any product that may be evaluated in this article, or claim that may be made by its manufacturer, is not guaranteed or endorsed by the publisher.
