# Supplementary material for: The WHO-ITU MyopiaEd Programme: A Digital Message Programme Targeting Education on Myopia and Its Prevention
Source: Front Public Health. 2022 May 26;10:881889. doi: 10.3389/fpubh.2022.881889 (PMC9177978; doi:10.3389/fpubh.2022.881889)
Supplement: Supplementary file 1 [file Table_1.DOCX]

Supplementary file 1. A summary of outcomes of expert review and pre-testing of the MyopiaEd message libraries

**Expert review**

**The International Expert Group (IEG)**

The IEG comprising of experts in myopia and health behaviour change were gathered from all WHO regions. The group was responsible for reviewing the best available evidence, and providing guidance on the context, purpose, end users, key topic themes, and modality of the proposed MyopiaEd component programme.

Table 1. Members of the IEG

| Name | Affiliation |
| --- | --- |
| Rosie Dobson | University of Auckland, New Zealand. |
| Hannah Faal | University of KwaZulu Natal, South Africa, African Vision Research Institute, South Africa |
| Ian Flitcroft | Centre for Eye Research, Ireland |
| Kate Gifford | Myopia Profile Pty, Ltd, Australia |
| Mingguang He | University of Melbourne, Australia |
| Rajiv Khandekar | King Khaled Eye Specialist Hospital, Saudi Arabia |
| Kovin Naidoo | University of KwaZulu Natal, African Vision Research Institute, South Africa ; EssilorLuxottica; France |
| Matt Oerding | Global Myopia Awareness Coalition, USA |
| Kyoko Ohno-Matsui | Tokyo Medical and Dental University, Japan; |
| Christine Wildsoet | University of California, Berkeley, USA |
| James Wolffsohn | Aston University, UK; |
| Geordie Woods | Sightsavers, USA |
| Tien Wong | Singapore National Eye Centre, Duke-NUS Medical School, National University of Singapore, Singapore |
| Sangchul Yoon | Yonsei University, South Korea |

**Key changes made to the message libraries during the expert review process included:**

- Widening the audience for Group 1 to the general population who might be involved in the care of children at risk of developing myopia including healthcare workers and teachers.
- The addition of notes around references to contact lenses and other treatment options within messages where these are routinely available and accessible within the target population.
- The term ‘optometrist’ was replaced with the generic term ‘eye health professional’ in messages. It was recommended that during local adaptation, the name of the professional type most involved in myopia detection/management should replace the term ‘eye health professional’.
- Messages about life-style related risk factors were reinforced more frequently throughout he message libraries targeting carers of children.

**Pre-testing**

**Population characteristics**

A total of 13 pre-testing sessions were conducted with 16 members of the target population including five members of the general population involved in the care of children at risk of developing myopia (two parents, a teacher, a general practitioner, and a pre-school carer), three parents/primary carers of children with myopia, four adolescents with myopia and four adults with myopia. Participants were adults aged between 16 to 60 years, spoke English, resided in New Zealand, and consented to be involved in the pre-testing. Eleven (69%) of the pre-testing participants were female.

**Feedback received**

Overall, all pre-testing participants were positive about the MyopiaEd programme and messages and reported that they would likely sign up for the programme if it were made available to them. They reported the messages and terminology used were easy to understand. They reported that the facts about myopia were particularly useful and interesting and that the actionable messages, for example, taking breaks when doing near activities, were also preferred. Parents commented that although many of the messages were things they already knew, they were nice reminders and when they were related to their child’s future health or academic performance were very motivating.

**Specific feedback from the pre-testing included:**

- *Members of Group 1 end users, the General population* reported that messages around taking breaks from near work should be careful not to deter children from reading and should therefore be specific around how long they should take a break for.
- It was reported that messages in Group 1 were too simplistic for health professionals and were better suited for the general population or lay health workers.
- Members of *Group 2 end users*, *Parents of children with myopia* thought that messages reminding their children that their role models wear glasses too were important, as their children were all self-conscious about their glasses (at least initially).
- Parents reported that the messages would be good conversation staters with their child(ren).
- Member of *Group 3 end users,* *Adolescents with myopia* reported that they thought that the messages reminding them to wear their glasses would be particularly useful. They liked the reminders to visit an eye health professional and to be mindful using screens. They reported the references to role models would be particularly motivating as was references to their future.
- Member of *Group 4 end users,* *Adults with myopia* felt that they would want to seek out more information after some of the messages. They felt that it would be good to include links to specific local resources, eye health professionals and other relevant services for more information and support.
- Adults with myopia also reported that they would not want too many reminders to see an eye health professional as it might feel like marketing which would decrease engagement.

**Feedback on the duration and frequency of messaging:**

- Parents preferred messages at times when they were with their children, for example, after school or in the evenings, but would prefer to have a break over weekends.
- It was thought that spreading the messages over a longer duration (1 year) may be more useful considering most people visit their eye health professional yearly. In particular, *Group 1,* *the* *general population,* felt that messaging might be more valuable spread over a longer period as the child might start to develop signs of myopia more than 6 months after starting the programme.
- Across all groups, message frequency of 1-2 per week was preferred.
- Programmes should start with the messages with interesting information/facts to increase engagement.

**Key changes made to the message library as a result of the pre-testing:**

- The *Group 1 end users, the General population* sub library algorithm was changed to deliver the messages over a 1-year period.
- The initial frequency of messaging was reduced across all sub-libraries.
- Messaging within sub-libraries were reordered to ensure that the interesting/engaging information was presented first and reminders later.
- Within the *Group 2 end users, ‘Parents/caregivers of children with myopia’* sub library messages to address children who are self-conscious about wearing glasses were added.
- Within the *Group 3 end users,* ‘Adolescent’ sub-library, additional reminders to wear their glasses and further references to role models and the future were added.
- Further guidance around the tailoring of messaging was added to guide the local adaptation of the libraries (e.g., adding of links to local services).
